# The relationship between interhemispheric homotopic functional connectivity and left-right difference of intrahemispheric functional integration in the human brain

**DOI:** 10.1162/imag_a_00205

**Published:** 2024-06-26

**Authors:** Xinhu Jin, Xinyu Liang, Gaolang Gong

**Affiliations:** CAS Key Laboratory of Behavioral Science, Institute of Psychology, Chinese Academy of Sciences, Beijing, China; State Key Laboratory of Cognitive Neuroscience and Learning & IDG/McGovern Institute for Brain Research, Beijing Normal University, Beijing, China; Institute of Science and Technology for Brain-inspire Intelligence (ISTBI), Fudan University, Shanghai, China; Chinese Institute for Brain Research, Beijing, China

**Keywords:** lateralization, intrahemispheric functional integration, homotopic functional connectivity, resting state, task state, age

## Abstract

The brain comprises left and right hemispheres, with notable distinctions in intrahemispheric functional integration observed between homotopic regions of each hemisphere. Previous studies have shown these left-right differences may be induced by interhemispheric connectivity between homotopic regions. However, no research has comprehensively investigated the relationship between lateralization of intrahemispheric functional integration and interhemispheric homotopic functional connectivity in the resting state. Based on resting-state functional connectivity, we identified two brain functional organization properties named lateralization of intrahemispheric functional integration (LI) and interhemispheric homotopic functional connectivity (HoFC), hypothesizing the former was modulated by the latter in healthy individuals. Results showed a widespread significant negative correlation between LI and HoFC among the whole brain, which could be affected by age and task state presenting a still negative pattern but with weaker strength, especially in heteromodal regions. Furthermore, two mediation models showed that HoFC significantly mediated the age and brain state effect on LI, suggesting age and task state might influence lateralization of intrahemispheric functional integration via interhemispheric homotopic functional connectivity in adults. Lastly, these two intrinsic organization properties with different heritability together correlated with the general intelligence factor in an antagonistic manner. In summary, our findings offer important and valuable insight into functional lateralization, functional homotopy, and their relationship from the perspective of intrinsic functional architecture, together with influential factors such as age and task state. These results provide direct evidence to further understand the link between the left and right hemispheres of the human brain, along with the relation to cognitive functions.

## 1 Introduction

How the left and right hemispheres of the brain interact with each other is a fundamental issue of general interest in neuroscience. Despite the overall mirroring patterns, the two hemispheres show a great deal of differences in specific structure and function (Herve et al., 2013;Toga & Thompson, 2003). Using functional magnetic resonance imaging (fMRI) under various tasks, previous studies have demonstrated hemispheric difference or dominance in multiple functional domains, including language (Cai et al., 2013), emotion (Lindell, 2013), spatial attention (Thomas et al., 2014), face processing (Meng et al., 2012), and memory (Habib et al., 2003). Using resting-state fMRI (rs-fMRI), hemispheric lateralization of intrinsic activity and functional connectivity has also been investigated (Liu et al., 2009;McAvoy et al., 2016), indicating the lateralization patterns of hemispheric functional integration and segregation (Gotts et al., 2013;D. Wang et al., 2014).

Functional integration among neural units is one of the fundamental principles in brain organization, believed to play a critical role in cognitive processing (Alavash et al., 2015;Cohen & D’Esposito, 2016). According toRingo et al. (1994), brain volume constrains high-speed processes to intrahemispheric clustering in bulky brains, leading the intrahemispheric functional integration to become predominant in adults, especially for language-related regions (Friederici, 2011;Perani et al., 2011). To quantify intrahemispheric functional integration for a specific brain region, the total sum of resting-state functional connectivity (rsFC) strength between this region and others within the same hemisphere can be employed (Chen et al., 2019;Gotts et al., 2013). Intriguingly, this intrahemispheric functional integration also showed significant left-right hemispheric differences, which correlated with cognitive performances in language and visuospatial tasks (Gracia-Tabuenca et al., 2018;Joliot et al., 2016). Further studies have shown that regional lateralization of intrahemispheric functional integration is influenced by several factors, such as handedness and mental disorders (Jiang et al., 2019;Joliot et al., 2016). However, to date, the underlying mechanisms responsible for this particular functional lateralization within the brain remain largely unexplored.

Notably, interhemispheric connectivity has been identified as a crucial factor in the emergence of brain functional lateralization. Owing to physiological and computational constraints on interhemispheric neural transmission time between the left and right hemispheres, the emergence of functional lateralization in the brain has been hypothesized to be affected by the conduction delay of interhemispheric connectivity during evolution with increased brain size (Ringo et al., 1994). Previous studies have provided evidence supporting the significant role of interhemispheric structural connections in influencing intrahemispheric functional integration as well as its hemispheric lateralization. For example, experimental disconnection of the corpus callosum (CC) leads to increased intrahemispheric functional integration within each hemisphere in rhesus monkeys (O’Reilly et al., 2013). In humans, individuals with congenital absences of interhemispheric structural connectivity (e.g., the agenesis of CC) exhibit more symmetric brain patterns between two hemispheres (L. Zuo et al., 2017).

In addition to structural connectivity, interhemispheric functional connectivity is also deemed to relate to brain functional lateralization. Specifically, interhemispheric functional connectivity between homotopic regions across the left and right hemispheres, that is, homotopic functional connectivity (HoFC) (Jin et al., 2020), has been observed to emerge and mature earlier than other types of functional connectivity (Smyser et al., 2010;Zhang et al., 2019) and exhibit the strongest connectivity strength among the entire brain following a peculiar interior spatial distribution corresponding well with functional hierarchies (Salvador et al., 2005;Stark et al., 2008). Moreover, HoFC has been identified as a guide for correct functional callosal targeting during early brain development (Suárez et al., 2014). A study has reported significant correlations between HoFC and activation lateralization in a language task (Tzourio-Mazoyer et al., 2016), suggesting a contribution of HoFC to brain functional lateralization. However, a comprehensive investigation exploring the relationship between HoFC and the lateralization of intrahemispheric functional integration, in both the resting state and task state, is still lacking.

Collectively, we hypothesized that the left-right lateralization of intrahemispheric functional integration is affected by interhemispheric homotopic functional connectivity. To test this hypothesis, we used two large open datasets of healthy adults: the Human Connectome Project (HCP) and the Cambridge Centre for Ageing and Neuroscience (Cam-CAN) datasets. We calculated the lateralization of intrahemispheric functional integration via the laterality index (LI) and HoFC for each pair of homotopic regions for each participant by using rs-fMRI data, indicating functional lateralization and functional homotopy. The relationship between LI and HoFC was then thoroughly investigated across subjects, using multiple statistical models. Specifically, we expected a negative correlation between LI and HoFC based on prior findings (Tzourio-Mazoyer et al., 2016). Additionally, we examined how various factors such as age and brain states influenced LI, HoFC, and their relationship. Furthermore, we inspected the heritability of LI and HoFC as well as their relations, anticipating different heritability patterns. Finally, we explored how these two brain fundamental intrinsic organization properties (LI and HoFC) correlated with human cognitions.

## 2 Methods

### 2.1 Datasets

In the present study, we used the HCP S1200 dataset (Van Essen et al., 2013) and the Cam-CAN dataset (Shafto et al., 2014). These two datasets are detailed below.

#### 2.1.1 HCP dataset

The HCP S1200 release includes a total of 1,207 healthy adults. Each subject participated in two separate resting-state fMRI (rs-fMRI) scans conducted on 2 consecutive days, known as REST1 and REST2. The tasking-state fMRI (ts-fMRI) protocols included task paradigms in seven different domains: motor, language, gambling, emotion, working memory, social cognition, and relational processing. Two runs with left-to-right (LR) and right-to-left (RL) phase encoding directions were acquired for both rs-fMRI and ts-fMRI scans. All subjects provided written informed consent on forms approved by the institutional review board of Washington University in Saint Louis. For more details, consult the paper byVan Essen et al. (2013).

##### 2.1.1.1 Behavioral tasks

All subjects took the Edinburgh Handedness Inventory (Oldfield, 1971) and a battery of cognitive tests, whose measured subdomains and names include processing speed (Pattern Completion Processing Speed), working memory (List Sorting), episodic memory (Picture Sequence Memory), executive function/cognitive flexibility (Dimensional Change Card Sort), executive function/inhibition (Flanker Task), language/vocabulary comprehension (Picture Vocabulary), and language/reading decoding (Oral Reading Recognition) (see the full list of tests we used inTable S1of Supplementary Materials).

##### 2.1.1.2 MRI acquisition protocols

To maximize the compatibility, rs-fMRI and ts-fMRI scans were acquired with the same acquisition parameters on a 3T Siemens Skyra scanner: a 32-channel head coil; whole-brain echo-planar imaging (EPI) sequence; a multiband acceleration factor of 8; repetition time (TR) = 720 ms; echo time (TE) = 33.1 ms; flip angle (FA) = 52°; bandwidth = 2,290 Hz/Pixel; in-plane field of view (FOV) = 208 × 180 mm^2^; 72 slices, voxel size = 2 mm isotropic voxels. The frames per rs-fMRI run were 1,200, while the frames across the seven ts-fMRI runs varied from 176 to 405 (emotion: 176, relational processing: 232, gambling: 253, social cognition: 274, motor: 284, language: 316, and working memory: 405). High-resolution T1-weighted structural images were acquired using a magnetization-prepared rapid gradient echo (MPRAGE) sequence: TR = 2,400 ms; TE = 2.14 ms; FA = 8°; FOV = 224 × 224 mm^2^; inversion time (TI) = 1,000 ms; voxel size = 0.7 mm isotropic; generalized autocalibrating partially parallel acquisition (GRAPPA) acceleration factor = 2.

##### 2.1.1.3 Image pre- and postprocessing

The HCP minimal preprocessing pipeline for fMRI volume was applied to both rs-fMRI and ts-fMRI images, including gradient distortion correction, motion correction, EPI distortion correction, registration to the Montreal Neurological Institute (MNI) space, intensity normalization to a global mean, and masking out nonbrain voxels. To further remove non-neural spatiotemporal components, all rs-fMRI scans were further denoised through a process that paired independent component analysis (ICA) with the FIX (FMRIB’s ICA-based X-noiseifier) (Griffanti et al., 2014;Salimi-Khorshidi et al., 2014;Smith et al., 2013). For more details, see the paper byGlasser et al. (2013).

In our present study, we focused on the cognitive relevance of rsFC-based measures, so we adopted a conservative strategy to remove the effect of head movement as much as possible (Siegel et al., 2017). Several procedures were further performed on the preprocessed fMRI scans by using the GRETNA toolbox (J. Wang et al., 2015). First, only rs-fMRI scans were linearly detrended to minimize the effects of low-frequency drift (Lowe & Russell, 1999). For both rs-fMRI and ts-fMRI scans, a set of nuisance variables including the Friston 24-parameter model, the average white matter (WM), and cerebrospinal fluid (CSF) signals were then regressed out (Ciric et al., 2017). Finally, the rs-fMRI images were temporally bandpass filtered (0.01–0.1 Hz) to minimize the high-frequency physiological noise (Fransson, 2005), while the ts-fMRI scans were high-pass filtered (>0.01 Hz) (Cole et al., 2014). For more details on the supporting evidence and rationale of these steps, seeJ. Wang et al. (2015).

According to the HCP quality control process, all subjects were rated with notable issues of images (QC_Issue_Codes explained). We excluded subjects flagged with anatomical anomalies or instabilities in the head coil during scans. In addition, a set of subjects was excluded due to incomplete time points of scan or lack of either phase encoding run. Finally, 887 right-handed subjects (age: 28.7 ± 3.7 years, 22–37 years; females: 477; handedness >40) with all qualified fMRI scans were entered into our following analyses. The zygosity and parent identities determined using the genotyping data from blood and saliva samples were available (HCP restricted data). In our present study, there were 146 twin pairs (96 monozygotic twins with 35 siblings and 2 half siblings; 50 dizygotic twins with 24 siblings and 4 half siblings), 440 siblings, 7 half siblings, and 83 unrelated individuals.

#### 2.1.2 Cam-CAN dataset

The study also utilized the Cam-CAN Stage 2 dataset, which comprised 652 healthy subjects (age range, 18–88 years). These individuals are native English speakers with normal or corrected-to-normal vision and hearing and have no neurological disorders. The study was approved by the Cambridgeshire 2 Research Ethics Committee, United Kingdom. Informed consents were obtained from all subjects. For more details, seeShafto et al. (2014).

##### 2.1.2.1 MRI acquisition protocols

All scans were collected using a 3T Siemens TIM Trio scanner with a 32-channel head coil. The rs-fMRI scans were acquired using an EPI sequence: whole-brain coverage; 32 axial slices, slice thickness of 3.7 mm with an interslice gap of 20%; TR = 1,970 ms; TE = 30 ms; FA = 78°; FOV = 192 × 192 mm^2^; voxel size = 3 × 3 × 4.44 mm^3^; 261 frames. High-resolution T1-weighted structural image was acquired using a MPRAGE sequence: TR = 2,250 ms; TE = 2.99 ms; TI = 900 ms; FA = 9°; FOV = 256 × 240 × 192 mm^3^; voxel size = 1 mm isotropic; GRAPPA acceleration factor = 2.

##### 2.1.2.2 Image processing

Using the field-map images, we first applied the FUGUE tool (https://fsl.fmrib.ox.ac.uk/fsl/fslwiki/FUGUE) to correct the EPI distortion for rs-fMRI scans. The resultant rs-fMRI images were then processed using the GRETNA toolbox (J. Wang et al., 2015). In brief, the first 10 volumes were discarded to make sure the magnetization approached dynamic equilibrium and the subjects adapted to the scanner. The remaining volumes were corrected for time offsets between slices due to interleaved acquisition and realigned to the first volume. By utilizing the Statistical Parametric Mapping (SPM) toolbox (http://www.fil.ion.ucl.ac.uk/spm/software/spm12/), the T1-weighted image was segmented into gray matter (GM), WM, and CSF. Subsequently, the diffeomorphic anatomical registration through exponential lie (DARTEL) algorithm based on warping information derived from the segmentation procedure was employed to normalize the T1-weighted image to the MNI space, yielding a deformation field. The T1-weighted image was coregistered to the rs-fMRI image, yielding another deformation field. The rs-fMRI images of each subject were then transformed to the MNI space via the combination of the two deformation fields above. In addition, normalized rs-fMRI images were further cleaned with the following steps: linearly detrending, regressing out the Friston’s 24 head motion parameters and average WM and CSF signals, and temporally bandpass filtering (0.01–0.1 Hz).

Notably, 64 subjects were excluded: 6 subjects due to lack of rs-fMRI or T1 scans, 4 subjects due to incomplete data acquisition (<261 volumes), 50 subjects due to severe head motion (displacement >2 mm, rotation >2°), 2 subjects due to the failure of spatial normalization to the MNI space, and 2 subjects due to lack of handedness information assessed by the Edinburgh Handedness Inventory (Oldfield, 1971). Finally, a total of 588 right-handed subjects (age: 54.0 ± 18.6 years; females: 300; handedness >40) were entered into our analyses.

### 2.2 Regional functional connectivity (FC)

To quantify FC at the regional level, the entire GM was parcellated into a set of biologically meaningful regions. Here, we used the atlas of intrinsic connectivity of homotopic areas (AICHA;www.gin.cnrs.fr/en/tools/aicha), which is designed for a finer delineation of functional homotopy based on rs-fMRI data and is putatively advantageous in analyses of brain hemispheric lateralization (Joliot et al., 2015). This atlas parcellates the GM into 172 pairs of cortical and 20 pairs of subcortical homotopic regions. We used 190 pairs of homotopic regions in AICHA after atlas refinement (seeSupplementary Materialsfor details).

#### 2.2.1 Resting-state FC

The Pearson correlation (*r*) was computed between the BOLD mean time series of two brain regions in the resting state, resulting in an rsFC matrix for each subject. To improve the normality, we performed Fisher’s*r*-to-*z*transformation on all rsFC values. Specifically in the HCP dataset, the two rsFCs (*z*) derived from the LR and RL phase encoding rs-fMRI runs in each session were further averaged as the final rsFC within each subject. While in the Cam-CAN dataset, no such rsFC average step was applied.

#### 2.2.2 Task background FC

In the present study, we focused on the FC of the task-general state (also referred to as background connectivity that exists independently of the trials of a task) (Elkhetali et al., 2019), rather than on the FC specific to any particular task state. As done previously with a similar approach (Cole et al., 2013,^2014^;Fair et al., 2007), this type of tasking-state FC should be estimated after suppressing or removing influences of (across-trial mean) task-related activations on task-related changes in FC. In our study, this removal was achieved by regressing the task events (any specific task effect of experiment design after convolution with the standard hemodynamic response function) from the BOLD series for each ts-fMRI run in the HCP dataset. The resultant residual BOLD series of all seven tasks were then concatenated after discarding the time points in fixation blocks (resting-state effect), putatively generating a series of task background activities (Cole et al., 2013,^2014^). The Pearson correlation (*r*) of the resultant concatenated time series between two regions was calculated and then Fisher’s*r*-to-*z*transformed. Finally, the FC values of the LR- and RL-phase encoding ts-fMRI runs in each session were averaged for each subject in the HCP dataset, resulting in the final task background FC (*z*).

### 2.3 Interhemispheric homotopic functional connectivity and lateralization of intrahemispheric functional integration

**Fig. 1.**
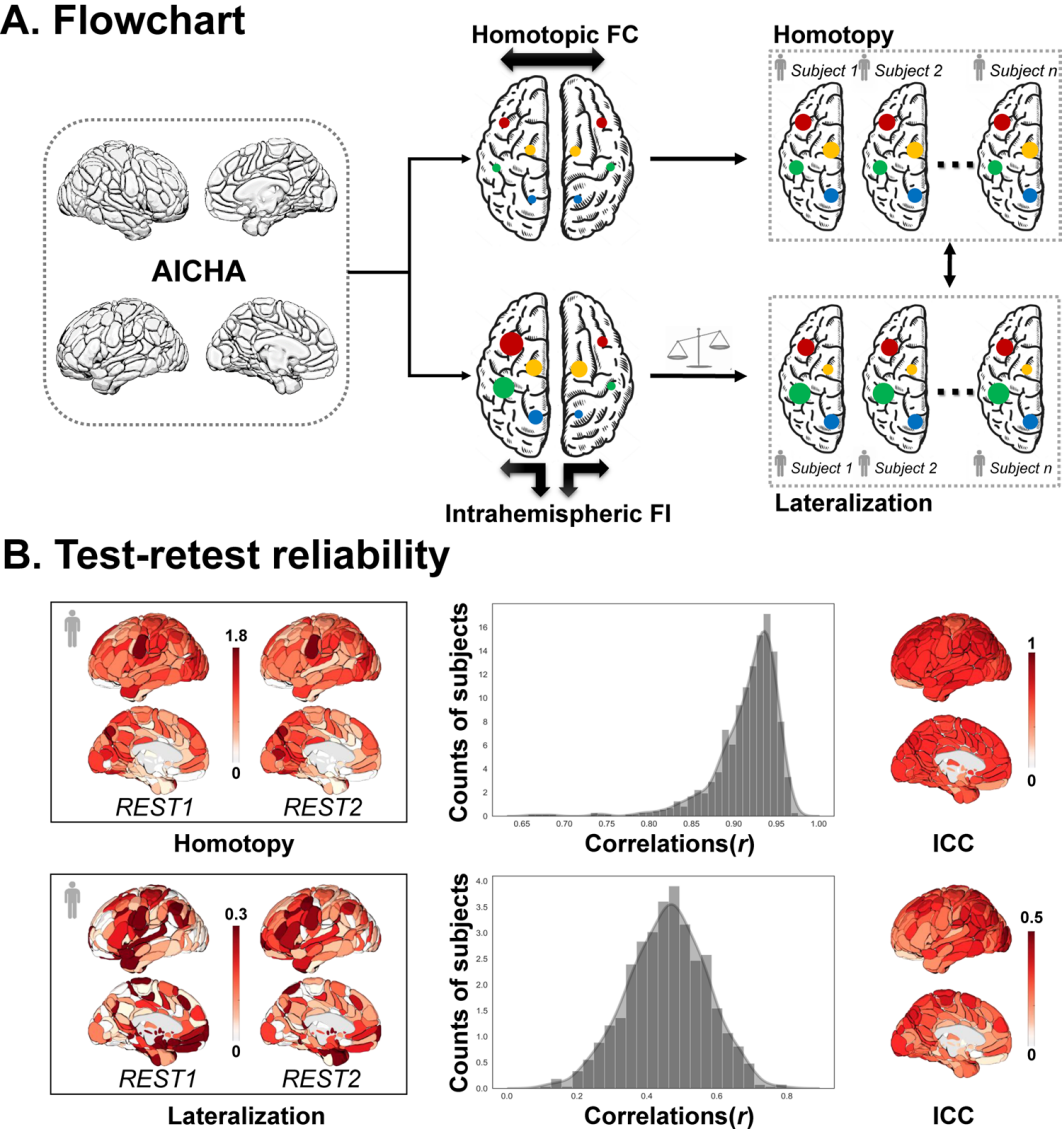
Frame of analyses. (A) Flowchart. For a specific brain region in the atlas of intrinsic connectivity of homotopic areas (AICHA), we first defined interhemispheric homotopic functional connectivity (HoFC) and intrahemispheric functional integration (intrahemispheric FI) illustrated by black arrows outside the brain. The former was calculated as the FC between this region and its homotopic region in the opposite hemisphere, and the latter was calculated as the sum of intrahemispheric FCs between this region and all the others within the same hemisphere. The color of the dots indicated their homotopic relationships, while the size of the dots represented the strength of HoFC and intrahemispheric functional integration. Then, two functional organization properties were defined for a pair of homotopic regions, named functional homotopy and functional lateralization. The former was defined as HoFC, whereas the latter was quantified by the laterality formula: LI = |(L - R)| / (|L| + |R|). (B) Test-retest reliability of HoFC (top) and LI (bottom) in the resting state in the HCP dataset. Left: The global maps of HoFC and LI in REST1 and REST2 of a randomly selected subject; middle: The individual pattern similarity distributions of HoFC and LI; right: The regional intraclass correlation coefficient (ICC) maps of HoFC and LI.

Based on the whole brain FC including both positive and negative correlations, we defined two brain functional organization properties. For a given pair of homotopic regions in AICHA, interhemispheric homotopic functional connectivity (HoFC) was simply calculated as the FC between one region and its contralateral homotopic counterpart (Fig. 1A).

The intrahemispheric functional integration of a specific region was calculated as the sum of FCs between this region and all the others within the same hemisphere. For each pair of homotopic regions in AICHA, the lateralization of intrahemispheric functional integration was calculated according to the following formula:

$$\text{L}{\text{I}}_{\text{i}}=\;|({\text{L}}_{\text{i}}-{\text{R}}_{\text{i}})|/(|{\text{L}}_{\text{i}}\text{​}|+|{\text{R}}_{\text{i}}\text{​}|).$$

This formula has been proven feasible and effective in previous research (Gracia-Tabuenca et al., 2018;Jansen et al., 2006;Westerhausen et al., 2014), where L_i_and R_i_represented the intrahemispheric functional integration of the left and right brain regions of homotopic pair*i*(Fig. 1A). Since the values of HoFC did not contain any directional information, we only focused on its relationship with the absolute degree of functional lateralization. LI values range from 0 to 1. For each pair of homotopic regions, a larger LI indicates a greater degree of functional lateralization, whereas a smaller LI suggests a tendency of functional symmetry.

### 2.4 Statistical analyses

#### 2.4.1 Test-retest reliability

To ensure the reliability and stability of the estimated functional properties, we first tested the reproducibility of LI and HoFC in the resting state. In this study, the REST1 scans in the HCP cohort were considered as the main resting samples for data analysis, and the REST2 scans as resting-retest samples. We calculated the Pearson correlations between these two samples across whole brain regions to obtain the overall pattern similarity distributions of HoFC and LI. At the regional level, we calculated the intraclass correlation coefficient (ICC) of HoFC and LI for each pair of AICHA regions.

#### 2.4.2 Relationship between LI and HoFC

To comprehensively explore the influence of functional homotopy on functional lateralization, we evaluated the partial correlation between LI and HoFC across subjects for each pair of homotopic regions in the resting state, controlled age, sex, handedness, and mean FC (mean of all possible FCs between any two regions in the whole brain). According to Mesulam’s functional hierarchy scheme (Mesulam, 1998), all brain regions belong to six different zones, for example, primary, unimodal, heteromodal, paralimbic, limbic, and subcortical (seeFig. S1in Supplementary Materials for the classification of regions based on AICHA). Therefore, we averaged values of the regions within the same hierarchical zone and further evaluated whether there were significant hierarchical differences in HoFC, LI, and their relationships by using the one-way analysis of variance (ANOVA).

#### 2.4.3 Canonical correlation analysis with multiple cognitions

The individual variations in connectivity patterns could be associated with certain cognitive performance. We hypothesized that the HoFC and LI would together contribute to potential cognitive benefits. Based on a range of cognitive measures collected in the HCP dataset, we employed canonical correlation analysis (CCA) to explore the relationship between brain functional organization properties and cognition. As a powerful multivariate method, CCA could capture associations across two modalities of data (e.g., brain and behavior) and provide significant correlations based on multivariate combinations of individual measurements. The CCA yielded a set of modes that demonstrated the maximum correlation between brain properties and cognitions. For each mode, we used 5,000 permutations to test the significance of the corresponding canonical correlation based on Bartlett’s approximate chi-squared statistic. The CCA loadings were calculated as the contributions of each original measurement to the identified latent variables. We used a customized script based on “canoncorr” function (Hotelling, 1992) implemented in Matlab.

In our study, a set of variables including the regional HoFC and LI in the resting state was individually residualized by a linear model including age, sex, handedness, and mean FC. The other set containing 24 cognitive measures (seeTable S1in Supplementary Materials for details) was also residualized by another linear model including age, sex, and handedness. All variables were standardized. Variables of brain (HoFC and LI of homotopic regions) and cognition (cognitive measures) with loadings over 0.3 are considered to be meaningfully contributive to visualization (Razavi et al., 2005;Thompson, 1984). To exhibit how two brain measures interacted with related specific CCA modes, we further correlated the loadings of HoFC and LI in the resting state.

#### 2.4.4 Heritability estimation

To test the influence of heritability on HoFC, LI, and their relationship in the resting state, we used the Sequential Oligogenic Linkage Analysis Routines (SOLAR) software package for the heritability estimation in the HCP dataset (Almasy & Blangero, 1998). All phenotypes of regional HoFC, regional LI, and their correlations were residualized with SOLAR for the available covariates, including age, sex, their interactions (age^2^, age × sex, age^2^× sex), and handedness. The univariate variance component method was used to evaluate the heritability of regional LI and HoFC, where the phenotypic variance of each brain property was decomposed as the sum of its additive genetic and environmental variance components (Almasy & Blangero, 1998). The bivariate variance component model was used to estimate the genetic correlations between all pairwise combinations of regional LI and HoFC (Almasy et al., 1997). This method decomposed the covariation between two phenotypes into a portion due to shared genetic factors and another portion due to shared environment. The genetic correlation measured the extent to which regional LI and HoFC were affected by shared genetic effects. These analyses were performed separately, using the pedigree kinship matrix derived from Solar and each of the empirical kinship matrices according to HCP demographic data. In addition, we averaged heritability values of regions belonging to the same zone of hierarchy and evaluated whether there were significant hierarchical differences in heritability between zones by using one-way ANOVA.

#### 2.4.5 Age effect and mediation analysis

To investigate age-related changes in LI and HoFC in the resting state, we used subjects from the Cam-CAN dataset and divided them into three groups (young: 18–39 years, middle: 40–59 years, and old: 60–87 years). We evaluated their regional relationships across subjects in each group, considering age, sex, handedness, and mean FC as confounding variables. To examine age effects, we used general linear model (GLM) analyses aimed at detecting regional differences in LI and HoFC in the developmental trajectory.

In addition, to find whether the HoFC can play a mediating role in the age-related effects on LI, we applied a standard step-wise mediation analysis by taking the age, HoFC, and LI as the predictor, mediator, and outcome, respectively. Sex, handedness, and mean FC were controlled as confounding variables. In this regard, this conventional test included the following four steps: (1) path*c*: the age effect on LI; (2) path*a*: the age effect on HoFC; (3) path*b*: the correlation between HoFC and LI, after controlling for the age factor; and (4) the indirect effect (a*× b*). If all tests of the four steps reached the level of significance, HoFC was regarded to significantly mediate the age effect on LI. The remaining a × b indirect effect was evaluated using the PROCESS macro implemented in SPSS (Hayes, 2018), with its confidence intervals by bootstrap method with 10,000 repetitions. An empirical 95% confidence interval that did not include 0 indicated significance at the 0.05 level. To reduce the times of multiple comparison, we combined the regions as several masks according to their correlation patterns. In each mask, all of the possible paths should be significant and identical in correlation direction across regions. We obtained the average values in candidate masks and further tested the significance of potential mediation pathways.

#### 2.4.6 Task effect and mediation analysis

To reveal the influence of brain state on the relationship between LI and HoFC, we also evaluated HoFC and LI as well as their relationship in the task state. To find relational differences between the resting state and task state, the Fisher’s*z*test was employed for comparing correlations between HoFC and LI. Besides, we compared LI and HoFC between two brain states using paired t-tests across subjects.

Based on the results above, to determine whether the HoFC can play a mediating role in the effects of different brain states on LI, we applied a two-condition within-participant mediation analysis using MEMORE macro in SPSS (Montoya & Hayes, 2017), with the resting/task state, HoFC of two brain states, and LI of two brain states as the predictor, mediator, and outcome, respectively, along with age, sex, handedness, and mean FC as confounding variables. For simplicity, we still used the standard step-wise convention for all candidate masks, including four-step tests: (1) path c: the differences of LI between the resting and task states, that is, the total effect of the predictor on the outcome; (2) path a: the differences of HoFC between the resting and task states; (3) path b: the correlation between the state-related alteration of HoFC and alteration of LI; and (4) the a × b effect, which was referred to as the indirect effect and was indicative of whether the predictor-outcome relationship was significantly reduced after controlling for the mediator. A bootstrap method with 10,000 repetitions was used to estimate the confidence intervals for the indirect effects. An empirical 95% confidence interval that did not include 0 indicated significance at the 0.05 level.

### 2.5 Validation analyses

To test whether the choice of parcellation could influence our main findings, we repeated our analyses above based on the Brainnetome Atlas (BNA;http://atlas.brainnetome.org/), which parcellates the GM into 105 pairs of cortical and 18 pairs of subcortical homotopic regions (Fan et al., 2016).

Unless otherwise mentioned, all the analyses in the resting state in the HCP dataset were conducted on REST1 scans. The statistics of correlations and group comparisons were performed using the SurfStat toolbox (https://www.math.mcgill.ca/keith/surfstat/) implemented in Matlab. All the results in our analyses were supposed to be significant with a*p*-value below 0.05 after Bonferroni correction or Games-Howell correction (with heterogeneity of variance). The regional results were projected to the surfaces and depicted by Surf-Ice software (https://www.nitrc.org/projects/surfice/). The point plots, histograms, and violin plots were generated by Seaborn package in Python (https://seaborn.pydata.org/).

## 3 Results

### 3.1 Test-retest reliability of HoFC and LI in the resting state

For the same subject in the HCP dataset, the global patterns of LI and HoFC were largely consistent between REST1 and REST2 scans. The individual pattern similarity distribution of HoFC showed a very high correlation across sessions, with a mean value of 0.92. While for LI, the individual pattern similarity distribution values were around 0.5, showing a medium level of similarity. The regional ICC of HoFC (mean values = 0.65, std = 0.11) also confirmed a widespread and moderately consistent pattern across the whole brain. The regional ICC of LI (mean values = 0.27, std = 0.09) was not as good as HoFC, but the values in occipital and parietal regions could reach a medium consistency (Fig. 1B). All these results indicated a modest but acceptable test-retest reliability of functional homotopy and functional lateralization.

### 3.2 Group average of two properties and their relationship in the resting state

**Fig. 2.**
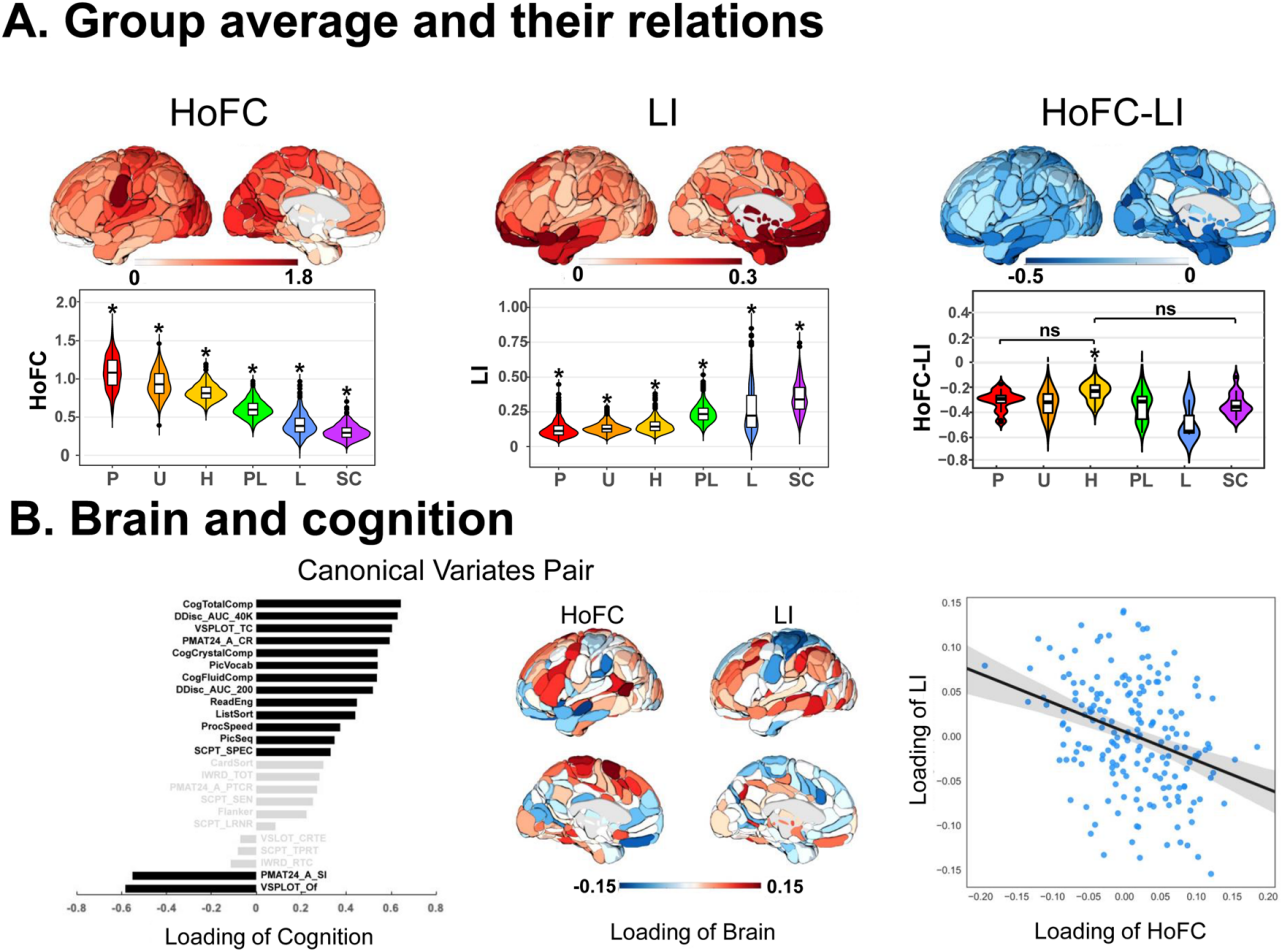
HoFC and LI in the resting state related to cognitions in the HCP dataset. (A) Group average of HoFC, LI, and their relation (HoFC-LI). The global group average and hierarchical subdivisions of HoFC and LI as well as their relation. P: primary, U: unimodal, H: heteromodal, PL: paralimbic, L: limbic, SC: subcortical. *Games-Howell-corrected*p*< 0.05 by post hoc tests between the hierarchical zone and the others. ns: no significance between two hierarchical zones. (B) Brain and cognition by canonical correlation analysis. Left: Loading of cognition in canonical variates pair (cognition loadings over 0.3 in black and under 0.3 in gray); middle: loading of brain regional HoFC and LI in canonical variates pair; right: the scatter plots between loading of HoFC and loading of LI.

In the HCP dataset, the regional HoFC and LI in the resting state showed different patterns at the group level (Fig. 2A). High HoFC values were observed in primary regions, such as visual, motor, and somatosensory areas, while low HoFC values were found in the prefrontal cortex, inferior temporal gyrus, and subcortical regions. In terms of LI, regions with strong lateralization in intrahemispheric functional integration were predominantly located in the frontal lobe, particularly the orbitofrontal cortex, extending to the temporal lobe as well as limbic and subcortical regions. The relationship between HoFC and LI showed a widespread significant negative pattern, where the strongest negative correlations were found in the lateral orbitofrontal cortex, medial temporal lobe, and cuneus.

The region-averaged HoFC, LI, and their relationship for subjects in each hierarchical zone are displayed in the violin maps. One-way ANOVA results revealed significant differences between hierarchical zones in both LI (Welch*F*= 1,033.88,*p*< 0.001) and HoFC (Welch*F*= 4,513.04,*p*< 0.001). Post hoc analyses revealed significant differences in any pairwise comparison of LI and HoFC among the six hierarchical zones. Specifically, HoFC exhibited a noticeable decrease from primary to subcortical regions, whereas LI displayed an opposite trend. Regarding the significant hierarchy difference in the relationship between HoFC and LI (Welch*F*= 9.10,*p*< 0.001), post hoc analysis revealed that only heteromodal regions demonstrated a significantly weaker negative correlation compared with regions in other hierarchical zones, except for primary and subcortical regions (Fig. 2A).

### 3.3 The link between cognitions and two brain properties in the resting state

To investigate whether the two brain properties have cognitive benefits, we performed CCA to link HoFC and LI with 24 cognitive measures in the HCP dataset. The CCA detected a single significant mode (canonical correlation*r*= 0.79,*p *< 0.001 by permutation test), which was strongly positively linked to the general intelligence factor (including fluid and crystal intelligence, self-regulation/impulsivity, spatial orientation processing, language/vocabulary comprehension, language/reading decoding, pattern comparison processing speed, working memory, picture sequence memory), and strongly negatively associated with cognitive failure (total skipped items of Penn Progressive Matrices and total positions off for all trials of Variable Short Penn Line Orientation Test). We displayed the loadings of LI and HoFC associated with this CCA mode. The mode showed positive associations with HoFC in some regions (including supplementary motor area, temporal cortex, inferior frontal gyrus, precentral sulcus, and cingulate sulcus) and positive associations with LI in some regions (including inferior parietal gyrus, superior and middle frontal gyrus, middle and inferior temporal gyrus, supramarginal gyrus, and precuneus). Post hoc analysis revealed a significant negative correlation between loadings of HoFC and loadings of LI across regions (*r*= -0.33,*p *< 0.001), suggesting that these two brain intrinsic organization properties played opposite roles in relation to cognitive functions (Fig. 2B).

### 3.4 Heritability of two properties and their relationship in the resting state

**Fig. 3.**
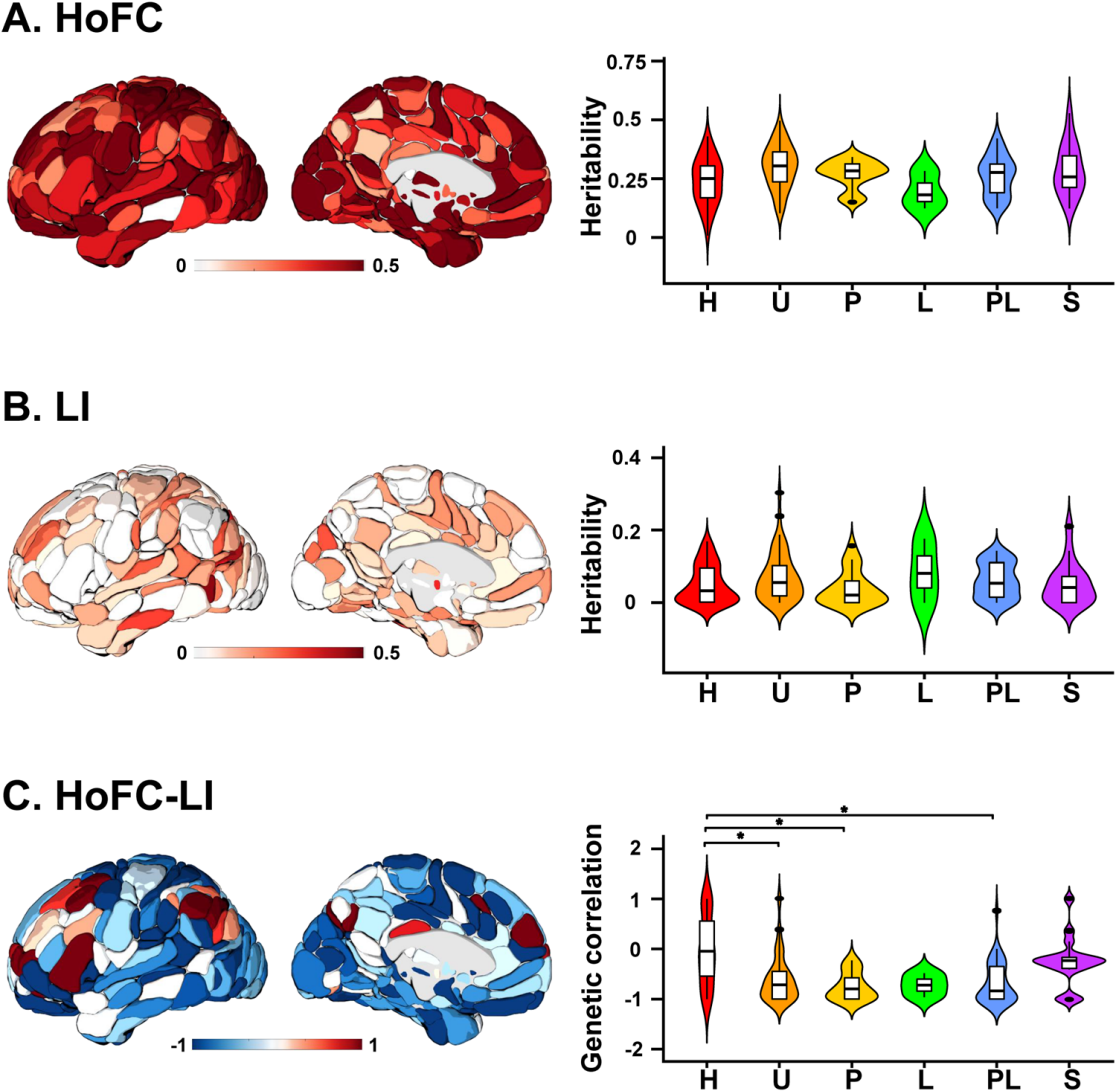
Heritability of HoFC and LI as well as their genetic correlation in the resting state in the HCP dataset. The global regional and hierarchical subdivisions of heritability of HoFC (A), LI (B), and their genetic correlation (C). P: primary, U: unimodal, H: heteromodal, PL: paralimbic, L: limbic, SC: subcortical. *Games-Howell-corrected*p*< 0.05 by post hoc tests between two hierarchical zones of regions after one-way analysis of variances.

The heritability map of HoFC revealed extensive regions with high heritability across the entire brain (Fig. 3A), most of which were significant after Bonferroni correction. The unimodal and subcortical regions exhibited higher heritability compared with other hierarchical zones of regions, although no significant difference was found in any pairwise comparison. In contrast, the heritability map of LI showed a different pattern (Fig. 3B). Significant heritabilities were only observed in the inferior temporal gyrus and middle occipital gyrus after Bonferroni correction. No significant difference in the heritability of LI was discovered across the regions of six hierarchical zones. When examining the regional genetic correlation between HoFC and LI, a widespread negative pattern was observed throughout the brain. However, significant negative genetic correlations were only identified in the posterior insula and paracentral lobule after Bonferroni correction. Additionally, the negative genetic correlations in heteromodal regions significantly differed from those in primary, unimodal, and paralimbic regions (Welch*F*= 8.49,*p*< 0.001,Fig. 3C).]

### 3.5 Age effect on two properties and their relationship in the resting state

**Fig. 4.**
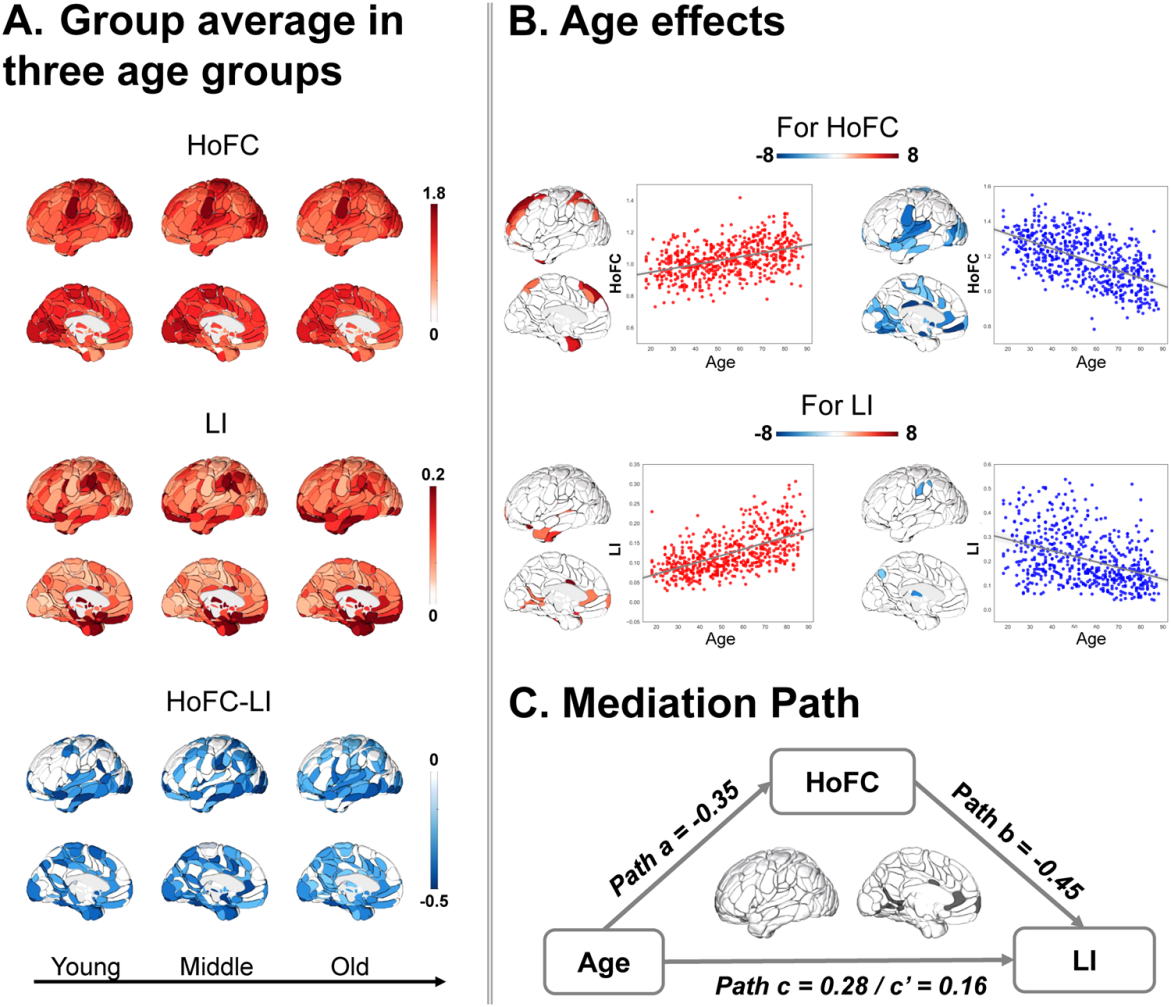
Age effect on HoFC and LI as well as their relationship (HoFC-LI) in the resting state in the Cam-CAN dataset. (A) The global group average of HoFC and LI as well as their relationship in three age groups (young: 18–39 years, middle: 40–59 years, old: 60–87 years). (B) Age effects on HoFC and LI. The age-related increase is depicted in red while the age-related decrease is depicted in blue. (C) Mediating pathways from the age to HoFC to LI in the mask covering the lingual gyrus, cingulum, parieto-occipital sulcus, posterior insula, and thalamus.

In the Cam-CAN dataset, the averaged global maps of HoFC and LI as well as their relation in the young group resembled those in the HCP dataset, in which subjects have a matched age range. Although the global patterns of HoFC and LI were highly consistent across the three age groups, we did observe age-related changes in some regions. Furthermore, we found a negative correlation between LI and HoFC in all three age groups, while the strength was weakened with age increasing (Fig. 4A).

To further investigate the developmental trajectories of regional HoFC and LI along with age, linear regression analyses were performed, with sex, handedness, and mean FC controlled. Results revealed that age-related increases of HoFC were located in the lateral and medial superior frontal cortex, temporal pole, superior parietal sulcus, and subcortical regions (hippocampus, thalamus, and caudate). In contrast, age-related decreases of HoFC were observed in the cuneus, precuneus, cingulum, fusiform gyrus, lingual gyrus, superior temporal cortex, parahippocampal gyrus, occipital lobe, and subcortical regions (caudate, putamen, thalamus, and amygdala). Regarding LI, positive linear relationships were found in the anterior and middle cingulum, lateral temporal pole, superior temporal sulcus, lingual gyrus, fusiform gyrus, and parieto-occipital sulcus. Conversely, negative linear relationships were observed in the supramarginal gyrus, precuneus, pallidum, and thalamus. To visualize the correlation, we combined all significant brain regions and averaged their values. We applied scatter plots to show the age effects on significant brain regions across subjects (Fig. 4B; for HoFC, mean positive effect: β = 0.51,*p*< 0.001, mean negative effect: β = -0.50,*p*< 0.001; for LI, mean positive effect: β = 0.56,*p*< 0.001, mean negative effect: β = -0.41,*p*< 0.001).

The significant mediation pathway (Fig. 4C) was found in a mask including the posterior insula, cingulum, lingual gyrus, parieto-occipital sulcus, and thalamus, in which HoFC can be considered a significant mediator of the age effect on LI (path c: β = 0.28,*p*< 0.001; path a: β = −0.35,*p*< 0.001; path b: β = −0.45,*p*< 0.001). The bootstrap simulation with 10,000 iterations further confirmed a significant indirect effect, with an effect size of c’ = 0.16 and a confidence interval of [0.12, 0.20].

### 3.6 Brain state effect on two properties and their relationship from the resting state to the task state

**Fig. 5.**
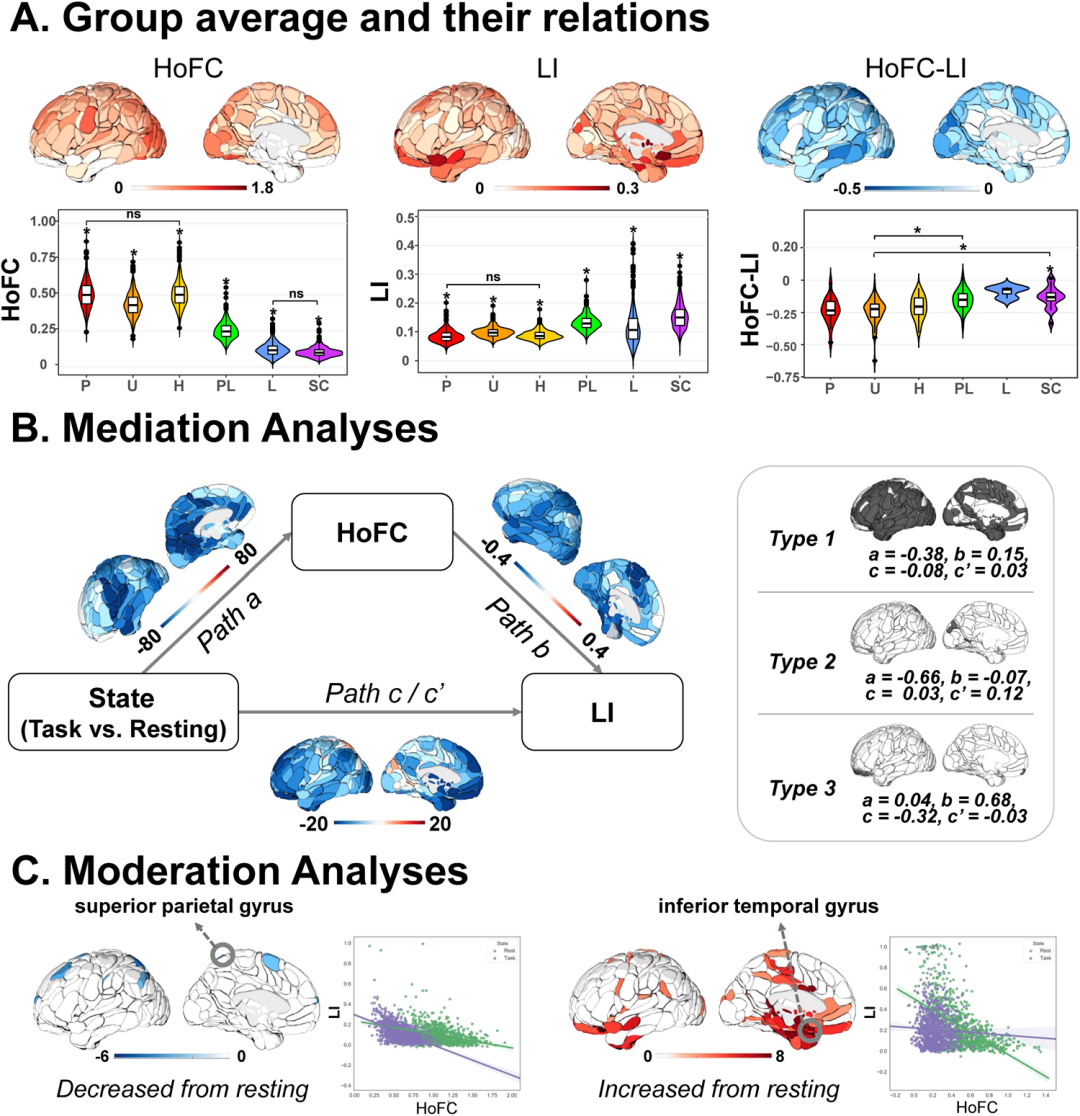
Task state effect on HoFC and LI as well as their relationship in the HCP dataset. (A) Group average of HoFC, LI, and their relationship (HoFC-LI). The global group average and hierarchical subdivisions of HoFC and LI as well as their relation in the task state. P: primary, U: unimodal, H: heteromodal, PL: paralimbic, L: limbic, SC: subcortical. *Games-Howell-corrected*p*< 0.05 by post hoc tests between the hierarchical zone and the others. ns: no significance between two hierarchical zones. (B) Mediating pathways from the brain state effect (task/resting) to HoFC to LI in regions with three different types. (C) The relationship between HoFC and LI from resting state to task state. The increased HoFC-LI relationship is depicted in red regions while the decreased HoFC-LI relationship is depicted in blue regions. The plots showed the relationship between HoFC and LI in two circled regions differing most in the HoFC-LI relationship (decreased most in the superior parietal gyrus and increased most in the inferior temporal gyrus) from resting state (green) to task state (purple).

In the task state, we observed that the global patterns of HoFC and LI as well as their relationships presented a lower strength than in the resting state. Moreover, the fundamental rules of hierarchy for both LI and HoFC also differed from those in the resting state. One-way ANOVAs revealed significant hierarchical differences in both LI (Welch*F*= 750.22,*p*< 0.001) and HoFC (Welch*F*= 7,551.03,*p*< 0.001). Post hoc analysis found significant differences between any pairwise comparison of the two properties, except the primary and heteromodal regions for both LI and HoFC. In addition, significant hierarchical differences for the HoFC-LI relationships also changed in the task state from the resting state (Fig. 5A).

To investigate whether the HoFC could mediate the brain state effect on LI, we conducted another mediation model, where the brain state was regarded as the predictor, and the HoFC and LI were the mediator and outcome, respectively. Utilizing the same approach as above, we found three different types of significant mediation pathways (Fig. 5B; type 1 in most cerebral cortex and subcortical regions: path c, β = -0.08,*p*< 0.001; path a, β = -0.38,*p*< 0.001; path b, β = 0.15,*p*< 0.001; type 2 in superior parietal gyrus, parieto-occipital sulcus, postcentral sulcus, lingual gyrus, cuneus: path c, β = 0.03,*p*< 0.001; path a, β = -0.66,*p*< 0.001; path b, β = -0.07,*p*< 0.001; type 3 in orbitofrontal cortex: path c, β = -0.32,*p*< 0.001; path a, β = 0.04,*p*< 0.001; path b, β = 0.68,*p*< 0.001). The 10,000 times bootstrap simulation further confirmed a significant indirect effect in all three types (type 1: c’ = 0.034 with confidence interval [0.025, 0.043]; type 2: c’ = 0.119 with confidence interval [0.101, 0.137]; type 3: c’ = -0.026 with confidence interval [-0.035, -0.018]).

To directly test the task effect on the relationship between HoFC and LI, we compared HoFC-LI relationships between the two brain states. The results demonstrated that most regions showed a greater degree of negative correlations between LI and HoFC in the resting state than in the task state. Compared with the resting state, the relationship between HoFC and LI in the task state showed a strength decrease in the supplementary motor region, postcentral sulcus, angular gyrus, superior frontal gyrus/sulcus, and superior parietal gyrus, while a strength increase in frontal regions, cingulum, cuneus, fusiform gyrus, lingual gyrus, supramarginal gyrus, temporal lobe, paracentral lobule, precuneus, parahippocampal gyrus, superior parietal gyrus, and subcortical regions (amygdala, hippocampus, thalamus, putamen, and caudate). Demonstration plots of two example regions (superior parietal gyrus/inferior temporal gyrus) showed different correlation patterns between HoFC and LI across two brain states (Fig. 5C).

### 3.7 Validation analyses

To verify the robustness of our main findings, we performed all analyses again by using a different brain parcellation (e.g., BNA). The results exhibited highly consistent findings with our main results on AICHA (seeFigs. S3–S6in Supplementary Materials).

## 4 Discussion

Based on a series of systematic and comprehensive analyses between lateralization of intrahemispheric functional integration and interhemispheric homotopic functional connectivity, our major findings were as follows. Firstly, our results demonstrated a widespread significant negative relationship between HoFC and LI in the resting state, which could be altered via the modulation by age and task state. Then on this basis, we verified the hypothesis that HoFC was considered to significantly mediate the age and brain state effect on LI. Lastly, these two brain intrinsic organization properties with different heritability together correlated with the general intelligence factor in an antagonistic manner.

### 4.1 The relationship between functional lateralization and functional homotopy in the resting state

Previous studies have explored the basic rules of interhemispheric homotopic functional connectivity and lateralization of intrahemispheric functional integration in the resting state, whose results were consistent with ours (Joliot et al., 2016;Stark et al., 2008;X.-N. Zuo et al., 2010). However, our work emphasized the relationship between these two brain organization properties, demonstrating a significantly strong negative correlation spread along the whole brain.Gracia-Tabuenca et al. (2018)defined HoFC and LI in the resting state using voxel-mirrored homotopic connectivity (VMHC) in school-aged subjects, but their findings were in agreement with ours. As the major neural pathway connecting homotopic cortical areas of the two brain hemispheres, CC plays a key role in mediating HoFC (Innocenti, 1986). Although subcortical areas might coordinate synchrony between bilateral cortical regions in a subject with a complete commissurotomy (Uddin et al., 2008), most studies have shown consistent results of interhemispheric coherence decreases in cases of CC agenesis or following callosotomy (Johnston et al., 2008;Owen et al., 2013;Roland et al., 2017). Moreover, interhemispheric functional connectivity can be predicted from the corresponding CC microstructure connecting homotopic regions (Mollink et al., 2019). The CC positively influences interhemispheric processing, promoting the integration of cerebral processing between both hemispheres and activating the unstimulated hemisphere (Geschwind & Galaburda, 1984). Therefore, Galaburda and Geschwind hypothesized that brains with more symmetry would exhibit stronger interhemispheric connections, suggesting that the development of asymmetry is associated with a lack of excitatory connections between hemispheres (Geschwind & Galaburda, 1984), which might directly relate to CC.Tzourio-Mazoyer et al. (2016)demonstrated a negative correlation between resting-state functional connectivity through the CC and activation lateralization in a language task. They proposed that intrinsic HoFC served as an indicator of the strength of excitatory activity and explained a moderate part of language lateralization variability across homotopic areas. Our findings with the same significant negative correlation between functional lateralization and functional homotopy in the resting state provided further evidence for the CC’s excitatory role in interhemispheric processing observed throughout the whole brain.

According to Mesulam’s hierarchical subdivision based on various studies in nonhuman primates and humans, sensory information undergoes elaboration and modulation along a core synaptic hierarchy as it integrates into cognition (Mesulam, 1998). In our study, the negative correlation between HoFC and LI in heteromodal regions was significantly weaker than in other hierarchical zones of regions. This result may be attributed to the middle level of heteromodal cortices in sensory-fugal processing. Served as neural bridges, these heteromodal regions link the inside to the outside world and allow multidimensional information integration. The connections between heteromodal regions and others are reciprocal and enable the binding of multiple unimodal and transmodal regions into distributed yet integrated multimodal representations (Mesulam, 1998). In line with this, our results also revealed that both HoFC and LI in heteromodal regions were moderate compared with other hierarchical zones of regions. Lower-level regions such as primary cortices engage in bilateral sensory integration and motor coordination, exhibiting stronger HoFC and weaker LI. While higher-level regions such as heteromodal, and paralimbic cortices operate more independently, displaying lower HoFC and stronger LI. Furthermore, our genetic correlations also showed a weaker negative correlation in heteromodal regions compared with others. These results suggested that the moderate intrinsic HoFC, LI, and their weak negative correlation in heteromodal regions may be influenced by genetic factors, highlighting the unique and vital role of heteromodal regions in information integration processes.

### 4.2 The changed relationship between functional lateralization and functional homotopy in the task state

In line with the works byCole et al. (2013,^2014^), we leveraged a general background pattern of functional connectivity across seven different tasks. This joint processing methodology provided a valuable foundation for comparing the two brain states, as it ensured a matched sample size of time points (Birn et al., 2013). Our results corroborated previous research findings indicating that functional connectivity patterns in the background during task performance were related to the intrinsic architecture observed in the resting state, albeit with weaker strength in widespread regions (Cole et al., 2014;Gratton et al., 2016;Lynch et al., 2018;Smith et al., 2009). We hypothesized that both inter- and intrahemispheric functional connectivity would undergo reorganization during the task state (Hermundstad et al., 2013), particularly in heteromodal regions, which could finally manifest in HoFC and LI as well as their relationships to fulfill diverse task demands (Cole et al., 2014). Generally speaking, our results revealed two different types of HoFC-LI relation alterations during task performance, in brain regions that have already been found to show task-related FC changes compared with the resting state (Lynch et al., 2018). In regions within the dorsal attention and frontoparietal networks (Yeo et al., 2011), characterized by involvement and cooperation in multiple tasks (Cole et al., 2013), the antagonistic interaction between HoFC and LI became much stronger to meet task demands. Conversely, in regions within the default-mode network and the limbic system (Yeo et al., 2011), which remain relatively inactive during task performance but active in the resting state (Dixon et al., 2017), the antagonistic interaction disappeared. Our findings reaffirmed the distinctions among task-positive and task-negative regions within distinct networks, offering a novel perspective on the altered interplay between brain functional organization properties during task engagement.

### 4.3 Functional homotopy mediates the effect of age and brain state on functional lateralization

On complete disconnection of the two hemispheres (anterior commissure and corpus callosum lesion),O’Reilly et al. (2013)discovered near-total abolition of functional connectivity between two brain hemispheres along with increasing correlations between regions within each hemisphere. This potential association indicates that homotopic functional connectivity may have an effect on intrahemispheric functional integration and further affects its lateralization. On the one hand, homotopic functional connectivity appeared and matured earlier than other types of functional connectivity (Smyser et al., 2010;Zhang et al., 2019). It could be first identified even in preterm infants aged as early as 26 weeks postmenstrual age (Smyser et al., 2010) and showed an increase during the majority of the first life year (Emerson et al., 2016). This increase in interhemispheric functional symmetry may serve as a general principle governing the development of multiple functional systems, including those that become lateralized in adulthood (Emerson et al., 2016). On the other hand, interhemispheric homotopic cortical activity is required for the correct targeting of CC, indicating that the establishment of structural connections between homotopic regions is essential for the bilateral integration of sensory and associative brain processing (Suárez et al., 2014). This bilateral integration through the CC excitation conduction may contribute to the lateralization of hemispheric integration. Furthermore, we observed widespread significant heritability of HoFC but hardly noticeable heritability of LI, suggesting that functional homotopy is more likely to be controlled by genetic factors than functional lateralization. Previous studies have provided evidence from multiple perspectives to support the notion that the genetic influence on brain traits follows the “symmetry principle,” whereby similar characteristics in the left and right brain hemispheres are controlled by the same genes or genomic regions, resulting in relatively higher heritability in the homotopic regions (McKay et al., 2013). Therefore, our heritability results also supported the assumption that interhemispheric homotopic functional connectivity could be an influencing factor for the lateralization of intrahemispheric functional integration in homotopic regions from the perspective of genetic time order.

Regarding the age effect, previous studies have already demonstrated an association between age and HoFC as well as LI in the regions we found inFig. 4C(Agcaoglu et al., 2015;X.-N. Zuo et al., 2010). They interpreted HoFC decreases and LI increases in these regions as evidence of increasing hemispheric specialization for higher-order cognitive processes, including memory encoding and retrieval (Banich & Brown, 2000). In terms of the brain state effect via mediation analysis, the most common type covering almost the whole brain regions meant both HoFC and LI decreased in the task state compared with the resting state, along with a positive correlation between differences of HoFC and differences of LI from the resting state to the task state. The rsFC differs from the transient coupling configurations of the active brain during task performance (Buckner et al., 2013;Hermundstad et al., 2013). Task modulation could affect both the interhemispheric and intrahemispheric connectivity (Hermundstad et al., 2013), thus leading to changes in HoFC and LI as well as their relation from the resting state to the task state.

However, these studies merely demonstrated that age and brain state were potential factors for functional homotopy and functional lateralization, without clarifying their specific relationship. Therefore, our mediation models are of great value in addressing two important issues: (1) How do age and brain state affect the two organization properties of interhemispheric homotopic functional connectivity and lateralization of intrahemispheric functional integration as well as their relationship? (2) Whether functional homotopy causes functional lateralization or vice versa? These questions are answered by our results that offer a more comprehensive understanding of how age and brain state impact functional organization property in the human brain and provide direct insights into the specific relationship between functional homotopy and functional lateralization.

### 4.4 The multivariate correlation between intrinsic brain organization properties and cognitions

Utilizing canonical analysis, we explored the multivariate associations among various brain regions and scores from diverse scales capturing a wide spectrum of cognitive dimensions. Notably, our investigation revealed a singular significant mode, illuminating the correlation between a specific pattern involving functional homotopy as well as functional lateralization and the general intelligence factor.Smith et al. (2015)identified a single significant CCA mode related to general intelligence based on the resting-state network derived from the HCP dataset. This congruence highlights the reliability of our CCA mode, corroborated by consistent findings across different regional parcellations. Examining the loadings of two intrinsic functional organization properties at the regional level, HoFC in sensory-motor, visual, prefrontal, and language-related regions, alongside LI in angular gyrus, superior temporal gyrus, middle frontal gyrus, and limbic regions, encompassing five functional hierarchical zones from primary to limbic, emerged as pivotal contributors to this mode. This suggested that successful cognitive functions rely on the cooperation and interaction of multiple brain regions, with their synaptic organization supporting both parallel and serial processing and allowing for multiple cognitive outcomes to be initiated by each sensory event (Mesulam, 1998). Furthermore, the observed negative correlation between the regional loadings of HoFC and LI in the resting state indicated an antagonistic process between the two brain organization properties once again, speculating that the negative relationship between functional homotopy and functional lateralization is a necessary pattern for successful cognitive functioning.

The present study has several limitations worth noting. Firstly, our utilization of two distinct public datasets came with varying imaging parameters. However, to mitigate potential confounds, we applied identical processing methodologies across both datasets, yielding consistent results among the two cohorts of young adults. Secondly, we refrained from regressing the effects of global signals, a contentious issue in the field. Prior studies have suggested that the global signal encapsulates hemispheric lateralization information, potentially bearing cognitive implications (McAvoy et al., 2016;Scholvinck et al., 2010). Therefore, we opted to use the coarse time series for constructing the functional connectivity matrix. Thirdly, our study employed two symmetric atlases, namely the AICHA (main results) and the BNA (validation results), to achieve our objectives. Although some regional disparities were observed between the outcomes obtained from these parcellation methods, the overarching trends of our primary findings remained robust and consistent. Finally, while our hypothesis was substantiated through correlation and mediation analyses, establishing causality requires more robust evidence. Future investigations should incorporate meticulously designed experiments and utilize appropriate statistical methodologies to facilitate more definitive conclusions regarding the causative relationship between functional homotopy and functional lateralization.

In summary, our study delved into the relationship between two brain fundamental organizational properties, namely functional lateralization and functional homotopy, uncovering a notable negative correlation across the entire brain in the resting state. Additionally, we examined influential factors such as age and task state, unveiling how they impact functional lateralization by modulating functional homotopy. Our findings yield crucial insights into the inherent functional architecture of the brain, shedding light on its interplay with age, task state, and heritability. These results furnish direct evidence contributing to a deeper understanding of the link between the left and right hemispheres in the human brain concerning cognitive functions.

## Supplementary Material

Supplementary Material

## Data Availability

The data used in this paper are available from the Human Connectome Project (HCP) dataset (https://humanconnectome.org/) and the Cambridge Centre for Ageing and Neuroscience (Cam-CAN) dataset (https://cam-can.org/). Data were analyzed using the codes uploaded on GitHub (https://github.com/liang-xinyu/Homotopy-IntraLI-Pipeline).
