# Supplementary material for: The relationship between interhemispheric homotopic functional connectivity and left-right difference of intrahemispheric functional integration in the human brain
Source: Imaging Neurosci (Camb). 2024 Jun 26;2:imag-2-00205. doi: 10.1162/imag_a_00205 (PMC12272220; doi:10.1162/imag_a_00205)
Supplement: Supplementary Material [file imag_a_00205-supp.pdf]

Supplementary materials

Atlas regions refinement

For either the atlas of intrinsic connectivity of homotopic areas (AICHA) or the Brainnetome Atlas (BNA), its parcellated region in the MNI space is not strictly confined to gray matter (GM) and may contain a small number of white matter (WM) voxels. To remove these possible WM voxels, we refined each region by using a GM mask generated from all HCP subjects in the MNI space. Specifically, the T1 image was segmented using the FreeSurfer pipeline (mri_aparc2aseg, https://surfer.nmr.mgh.harvard.edu/) for each HCP subject, resulting a cortical GM/WM mask in the MNI space. We calculated a group-level cortical probability map by averaging all subjects' cortical GM masks and then applied a threshold of 0.2, ending up with an initial group-level cortical GM mask. For subcortical nuclei, we further used the FIRST tool embedded in the FSL to segment out all subcortical structures (14 in total) for each subject. Likewise, a group-level probability map for each subcortical structure was generated and a threshold of 0.2 was applied. These resultant masks for subcortical structures were then merged into the initial group-level cortical GM mask, yielding a final high-quality GM mask. Notably, two pairs of AICHA subcortical regions (N_Caudate-2 and N_Thalamus-8) were excluded from our study, because they were not overlapped with the GM mask or had an extremely small number of voxels (less than 10). For BNA, all regions were reserved.

Table S1 Behavior tests used in the HCP dataset

Full Display Name	Assessment	Abbreviation
NIH Toolbox Picture Sequence Memory Test: Age-Adjusted Scale Score	Episodic Memory	PicSeq
NIH Toolbox Dimensional Change Card Sort Test: Age-Adjusted Scale Score	Executive Function/ Cognitive Flexibility	CardSort
NIH Toolbox Flanker Inhibitory Control and Attention Test: Age-Adjusted Scale Score	Executive Function/ Inhibition	Flanker
Penn Progressive Matrices: Number of Correct Responses	Fluid Intelligence	PMAT24_A_CR
Penn Progressive Matrices: Total Skipped Items	Fluid Intelligence	PMAT24_A_SI
Penn Progressive Matrices: Median Reaction Time for Correct Responses	Fluid Intelligence	PMAT24_A_RTCR
NIH Toolbox Oral Reading Recognition Test: Age-Adjusted Scale Score	Language/ Reading Decoding	ReadEng
NIH Toolbox Picture Vocabulary Test: Age-Adjusted Scale Score	Language/ Vocabulary Comprehension	PicVocab
NIH Toolbox Pattern Comparison Processing Speed Test: Age-Adjusted Scale Score	Processing Speed	ProcSpeed
Delay Discounting: Area Under the Curve for Discounting of $200	Self-regulation/ Impulsivity	DDisc_AUC_200
Delay Discounting: Area Under the Curve for Discounting of $40,000	Self-regulation/ Impulsivity	DDisc_AUC_40K
Variable Short Penn Line Orientation: Total Number Correct	Spatial Orientation	VSPLOT_TC
Variable Short Penn Line Orientation: Median Reaction Time	Spatial Orientation	VSPLOT_CRTE
Variable Short Penn Line Orientation: Total Positions Off for All Trials	Spatial Orientation	VSPLOT_OFF
Short Penn Continuous Performance Test: Median Response Time for True Positive Responses	Sustained Attention	SCPT_TPRT
Short Penn Continuous Performance Test: Sensitivity	Sustained Attention	SCPT_SEN
Short Penn Continuous Performance Test: Specificity	Sustained Attention	SCPT_SPEC
Short Penn Continuous Performance Test: Longest Run of Non-Responses	Sustained Attention	SCPT_LRNR
Penn Word Memory Test: Total Number of Correct Responses	Verbal Episodic Memory	IWRD_TOT
Penn Word Memory Test: Median Reaction Time for Correct Responses	Verbal Episodic Memory	IWRD_RTC
NIH Toolbox List Sorting Working Memory Test: Age-Adjusted Scale Score	Working Memory	ListSort
NIH Toolbox Cognition Fluid Composite: Age-Adjusted Scale Score	Cognition Fluid Composite	CogFluidCom
NIH Toolbox Cognition Total Composite Score: Age-Adjusted Scale Score	Cognition Total Composite Score	CogTotalComp
NIH Toolbox Cognition Crystallized Composite: Age-Adjusted Scale Score	Cognition Crystallized Composite	CogCrystalComp

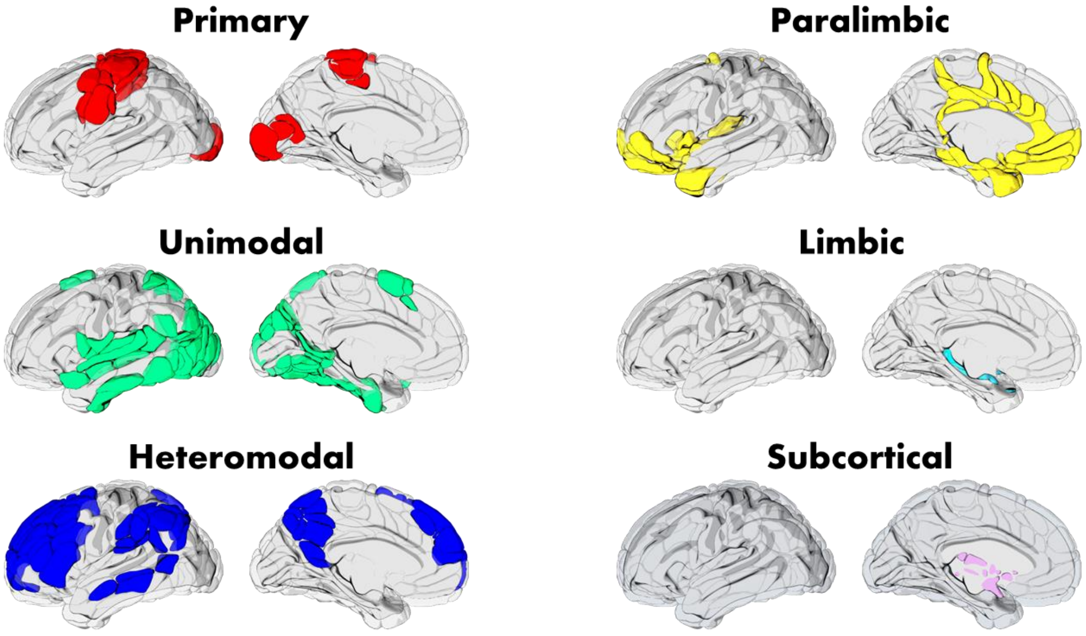

Fig. S1. Regional classification according to functional hierarchy in the atlas of intrinsic connectivity of homotopic areas (AICHA). Functional hierarchy groupings consisted of primary, unimodal, heteromodal, paralimbic, limbic, and subcortical. AICHA includes both regions located in the crown of the gyri (named Gyrus, region name beginning with “G_”) and regions located in the depth of the sulci (named Suclus, region name beginning with “S_”). The subcortical nuclei were labeled separately (name Nucleus, region name beginning with “N_”). Different parcels belonging to the same anatomical region were labeled with numbers (starting from 1).

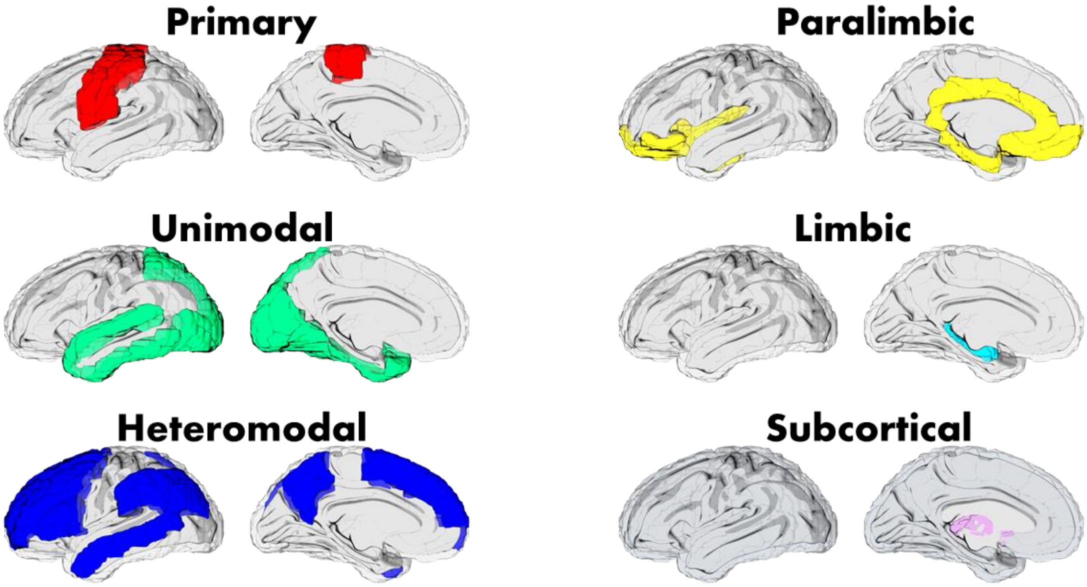

Fig. S2. Regional classification according to functional hierarchy in the Brainnetome Atlas (BNA). Functional hierarchy groupings consisted of primary, unimodal, heteromodal, paralimbic, limbic, and subcortical. In BNA, different parcels belonging to the same anatomical region were labeled with numbers (starting from 1).

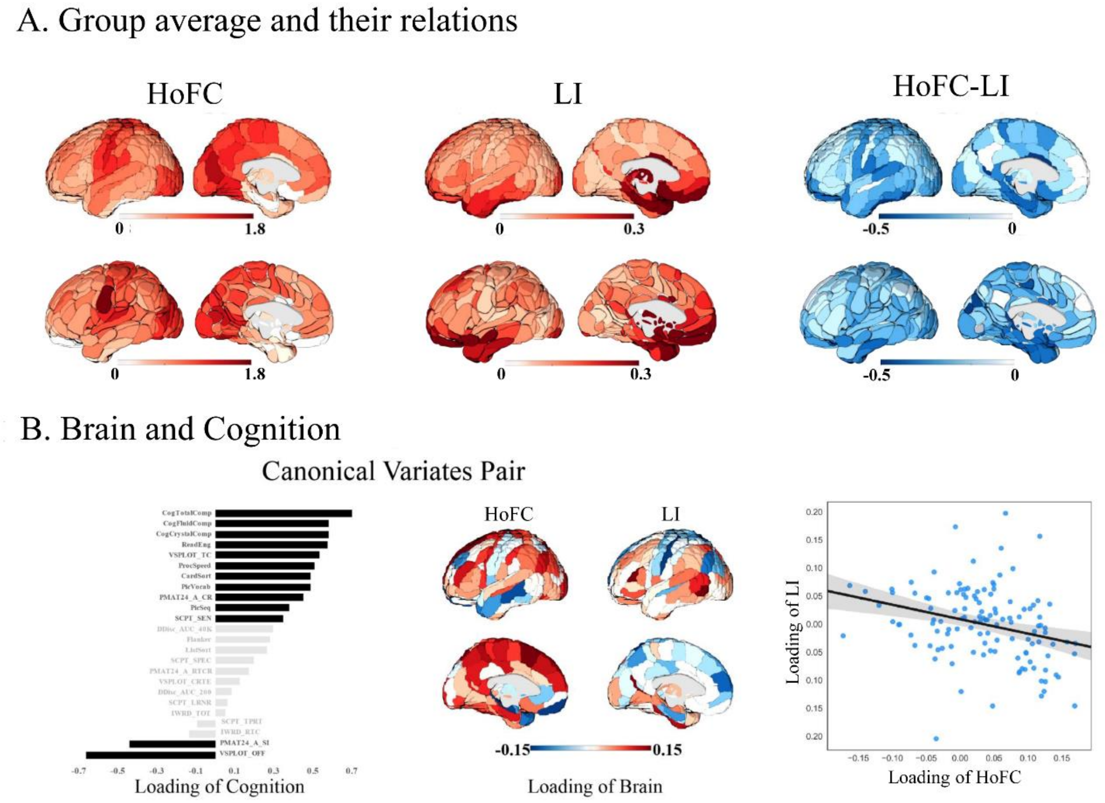

Fig. S3. HoFC and LI in the resting state related to cognitions in the HCP dataset. (A) Group average of HoFC, LI, and their relation (HoFC-LI). The global group average of HoFC and LI as well as their relation in REST1 based on BNA (top) and in REST2 based on AICHA (bottom). (B) Brain and cognition by canonical correlation analysis in REST1 based on BNA. Left: Loading of cognition in canonical variates pair (cognition loadings over 0.3 in black and under 0.3 in gray); Middle: Loading of brain regional HoFC and LI in canonical variates pair; Right: The scatter plots between loading of HoFC and loading of LI.

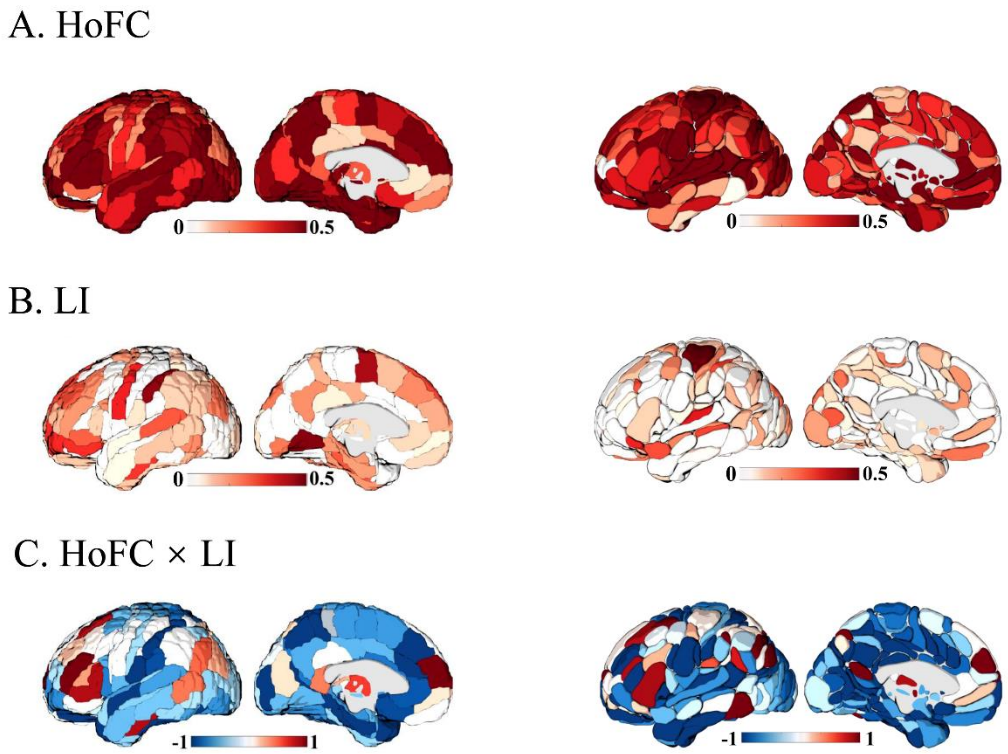

Fig. S4. Heritability of HoFC and LI as well as their genetic correlation in the resting state in the HCP dataset. (A) The global regional heritability map of HoFC (A), LI (B) as well as their genetic correlation (C). Left: REST1 scans based on BNA. Right: REST2 scans based on AICHA.

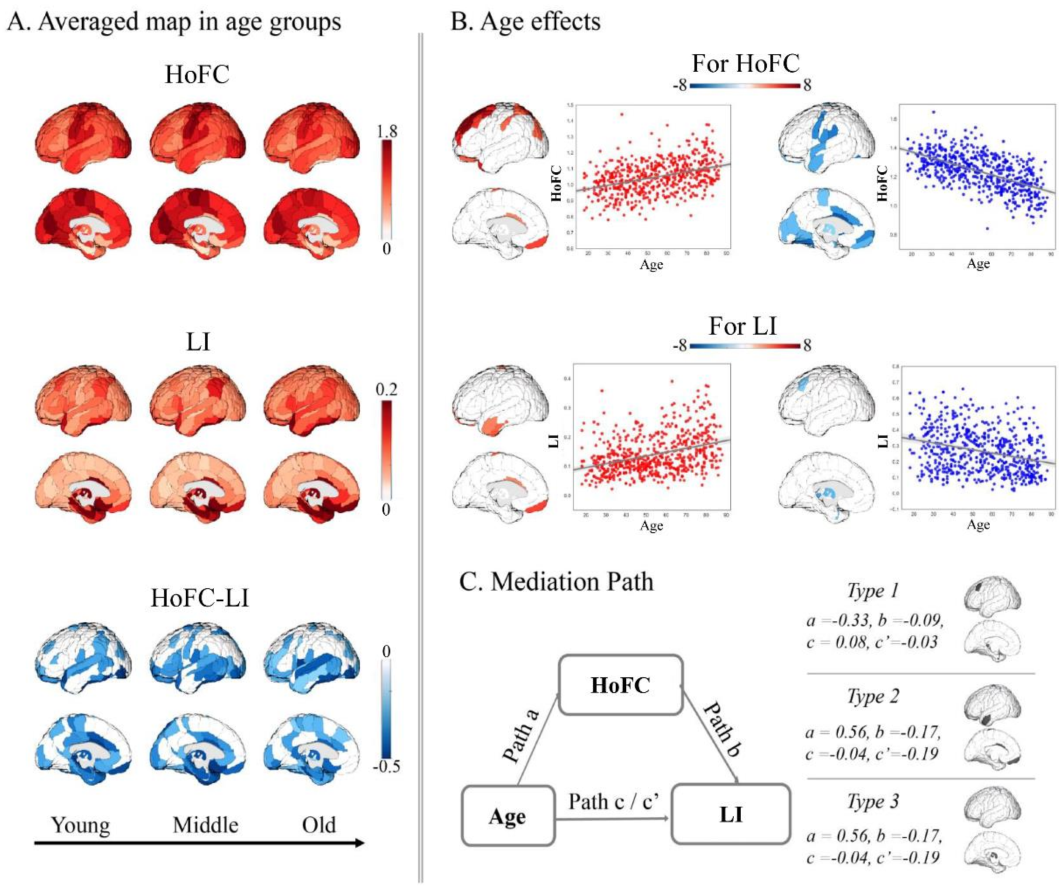

Fig. S5. Age effect on HoFC and LI as well as their relationship (HoFC-LI) in the resting state in the Cam-CAN dataset based on BNA. (A) The global group average of HoFC and LI as well as their relation in three age groups (young: 18-39 years, middle: 40-59 years, old: 60-87 years). (B) Age effects on HoFC and LI. The age-related increase was depicted in red while the age-related decrease was depicted in blue. (C) Mediating pathways from the age to HoFC to LI.

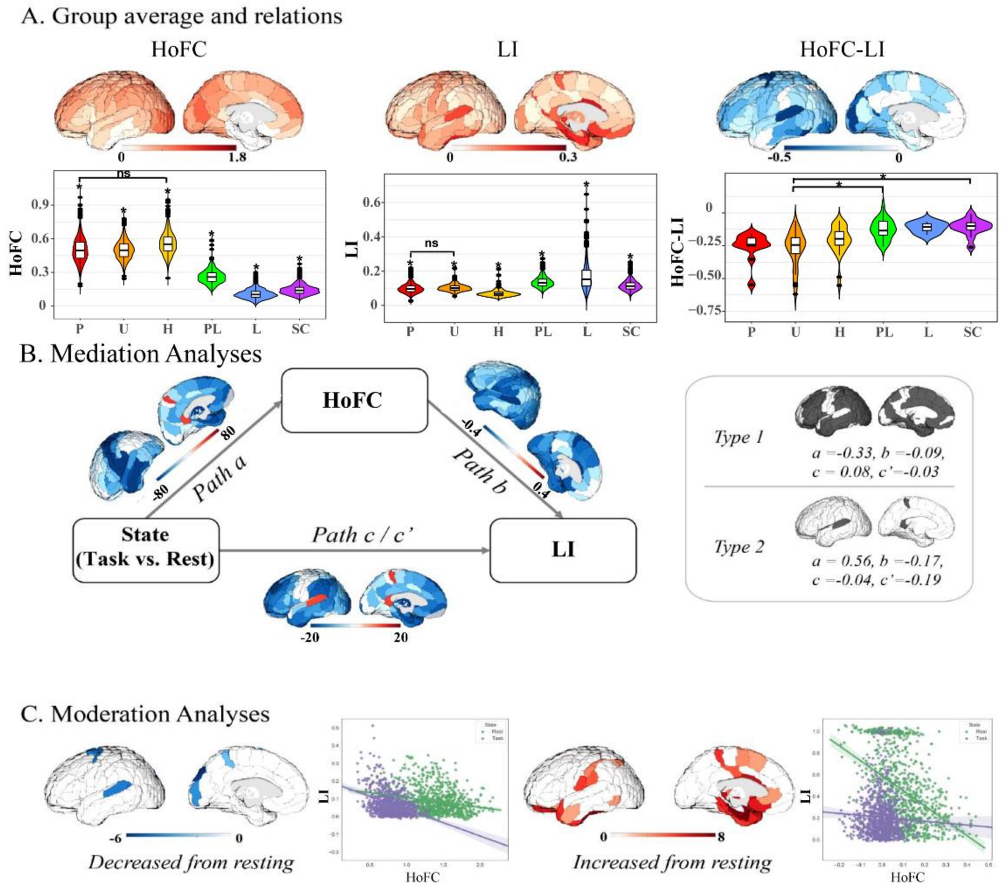

Fig. S6. Task state effect on HoFC and LI as well as their relationship in the HCP dataset based on BNA. (A) Group average of HoFC, LI, and their relation (HoFC-LI). The global group average and hierarchical subdivisions of HoFC and LI as well as their relation in the task state. P: primary, U: unimodal, H: heteromodal, PL: paralimbic, L: limbic, SC: subcortical. * Games-Howell-corrected $p < 0.05$ by post-hoc tests between the hierarchical zone and the others. ns: no significance between two hierarchical zones. (B) Mediating pathways from the brain state effect (Task/Resting) to HoFC to LI in regions with two different types. (C) The relationship between HoFC and LI from resting state to task state. The increased HoFC-LI relationship was depicted in red regions while the decreased HoFC-LI relationship was depicted in blue regions. The plots showed the relationship between HoFC and LI in two regions differing most in the HoFC-LI relationship from resting state (green) to task state (purple).
